# The Time of Lenten Lilies

**Published:** 1887-04-23

**Authors:** 


### THE TIME OF LENTEN LILIES.

By a Cumberland Lady.

THEY are dancing to-day, the daffodils, as Wordsworth saw them dancing long ago in the breeze, a countless ballet of gold and green coryphées, all garbed alike, but each one posed with infinite variety. And they dance upon a wondrous fair stage, one of Nature's handiwork, traversed by a river with rocky banks—a river that murmurs or brawls alternately, and finally winds away into the shadows of many-tinted hills. On one side there is a plateau of green meadow-land, shelving abruptly upward to the sky, and crowned with feathery larch and coppice woods. The year's young blood is stirring every twig; they wait with youth's impatience to burst their bronzy fetters, and give their tender colours to the spring. The other bank is wooded to the edge. Hazel and willow boughs, in amber and silvery grey, survey their reflected beauty in the river's face. At their feet among the boulders, in the grasses, on the upland and the crevices of rock, glow the daffodils. Spreading to right and left as far as sight can reach, a very wealth and gladness to the eyes. The earliest harvest of the northern year, of truer gold than e'er was bearded grain, and countless as the stars for multitude—where Wordsworth saw them on a dead spring day, and sang their praises with a poet's tongue. Here in this very spot he must have stood by the river his pen has made historical, bathed in the sun that gilds the earth to-day, and gives all shades of azure to the hills. Standing on ground like this, with the dainty "bell-roses" carpeting the place, one's mind naturally reverts a century or two, to where, in panoply of ancient splendour, England and France greeted each other's chivalry upon the plains of Ardres. And yet methinks, whatever magnificence of jewelled hilt and helmet, whatever shone gorgeous from the looms of rival empires, beneath the standards of Albion and sunny Gaul, where Henry of England and his royal brother met, must have been as naught compared with the riches round me now—loveliness unborn of man's devising—but mother Nature's gift—the magic touch of God's almighty finger upon this quiet valley where the river flows outward to the sea.

